# Bioreactor for mobilization of mesenchymal stem/stromal cells into scaffolds under mechanical stimulation: Preliminary results

**DOI:** 10.1371/journal.pone.0227553

**Published:** 2020-01-10

**Authors:** Carolina Gamez, Barbara Schneider-Wald, Andy Schuette, Michael Mack, Luisa Hauk, Arif ul Maula Khan, Norbert Gretz, Marcus Stoffel, Karen Bieback, Markus L. Schwarz

**Affiliations:** 1 Department for Experimental Orthopaedics and Trauma Surgery, Orthopaedics and Trauma Surgery Centre (OUZ), Medical Faculty Mannheim of the University of Heidelberg, Mannheim, Baden Württemberg, Germany; 2 Medical Research Centre (ZMF), Medical Faculty Mannheim of the University of Heidelberg, Mannheim, Baden Württemberg, Germany; 3 Institute of General Mechanics, RWTH Aachen University, Aachen, Nordrhein-Westfalen, Germany; 4 Institute of Transfusion Medicine and Immunology, FlowCore Mannheim, German Red Cross Blood Service of Baden Württemberg-Hessen, Medical Faculty Mannheim of the University of Heidelberg, Mannheim, Baden Württemberg, Germany; Università degli Studi della Campania, ITALY

## Abstract

**Introduction:**

Articular cartilage (AC) is a viscoelastic tissue with a limited regenerative capability because of the lack of vasculature. Mechanical stimulation contributes to the homeostasis of functional AC since it promotes the delivery of nutrients, cytokines and growth factors between the distant chondrocytes. We hypothesized that biomechanical stimulation might enhance mobilization of endogenous mesenchymal stem/stromal cells (MSCs) from neighboring niches as the bone marrow.

**Aim:**

This study aimed to introduce a bioreactor for inducing mobilization of MSCs from one compartment to another above by mechanical stimulation *in vitro*.

**Methods:**

A novel mechanical system for evaluating mobilization of cells in a 3D context *in vitro* is presented. The system consists of a compression bioreactor able to induce loading on hydrogel-based scaffolds, custom-made software for settings management and data recording, and image based biological evaluation. Intermittent load was applied under a periodic regime with frequency of 0.3 Hz and unload phases of 10 seconds each 180 cycles over 24 hours. The mechanical stimulation acted on an alginate scaffold and a cell reservoir containing MSCs below it. The dynamic compression exerted amplitude of 200 μm as 10% strain regarding the original height of the scaffold.

**Results:**

The bioreactor was able to stimulate the scaffolds and the cells for 24.4 (±1.7) hours, exerting compression with vertical displacements of 185.8 (±17.8) μm and a force-amplitude of 1.87 (±1.37; min 0.31, max 4.42) N. Our results suggest that continuous mechanical stimulation hampered the viability of the cells located at the cell reservoir when comparing to intermittent mechanical stimulation (34.4 ± 2.0% *vs*. 66.8 ± 5.9%, respectively).

Functionalizing alginate scaffolds with laminin-521 (LN521) seemed to enhance the mobilization of cells from 48 (±21) to 194 (±39) cells/mm^3^ after applying intermittent mechanical loading.

**Conclusion:**

The bioreactor presented here was able to provide mechanical stimulation that seemed to induce the mobilization of MSCs into LN521-alginate scaffolds under an intermittent loading regime.

## Introduction

Articular cartilage (AC) is a viscoelastic tissue with scarce number of chondrocytes and a rich organized extracellular matrix (ECM), composed mainly of type-II collagen and proteoglycans. This enables compressibility to support weight and to distribute loads of the joints [1]. The absence of blood and lymphatic vessels restricts the direct access to nutrients and limits healing capability [2]. In an avascular tissue where nutrients and cytokines are mainly distributed by diffusion, joint motion assists to the transport rate of solutes [3]. Mechanical activity in joints supports the proper functionality of chondrocytes, improves ECM content, promotes glycosaminoglycan synthesis, and fiber organization [4, 5]. Furthermore, mechanical stimulation contributes to the synthesis of ECM components by chondrocytes even if isolated from patients with osteoarthritis [6]. Thus, mechanical loading plays a pivotal role for the nutrients delivery, waste disposal, repair and AC healthiness [7].

Bioreactors are biotechnological devices used in cartilage engineering to evaluate diverse mechanical stimulation strategies as compression, tension, hydrostatic pressure and shear stress, acting on natural cartilage tissue or cartilage-like constructs [8–11]. Of these mechanical stimuli, direct compression has been widely examined, since it simulates the stress exerted on AC by the opposite joint component [12]. Compressive forces have been applied either under static or dynamic conditions, but dynamic stimulation mimics the physiological environment of the AC better. Furthermore, it has been shown to induce proteoglycan and collagen-II synthesis [13].

The standard clinical procedures for treating AC defects are autologous chondrocytes implantation (ACI), mosaicplasty, and microfracture. The current ACI therapy known as matrix-induced ACI (MACI), consists on isolating the chondrocytes in a first surgery, *in vitro* expansion for several weeks, cell implantation in a matrix, and its implantation in a second surgical procedure [14]. Mosaicplasty implies the use of osteochondral autologous plugs taken from non-weight bearing regions to transplant them in the defect area. Lastly, microfracture focuses on stimulating the bone marrow through micro-perforations in the subchondral bone, which promotes a blood clot formation in the defect site, containing progenitor cells and growth factors that induces defect healing. Despite their wide application, the clinical outcomes are not fully fulfilled yet, mainly because the repaired tissue is fibrotic with inferior quality biochemical and mechanically in comparison to hyaline cartilage [15].

To maintain tissue homeostasis in the body, stem and progenitors cells can be mobilized from their niches to the injury site for repairing [16]. Mesenchymal stem/stromal cells (MSCs) are target components for cell-based therapies, e.g., for bone and cartilage injuries, because of their multi-lineage differentiation potential and properties that promote regeneration [17]. Alternative to their application as *ex vivo* expanded cell products, induction of endogenous mobilization may provide a better outcome than current therapies. MSCs with chondrogenic differentiation potential reside in bone marrow, stroma, synovium, infrapatellar fat pad and periosteum [18, 19], of which the bone marrow has been the most common reservoir to obtain MSCs in cartilage defects research [20]. Although, AC on the joint is spatially separated from the bone marrow by the subchondral bone lamella.

We hypothesize that mechanical stimuli are involved in endogenous cartilage repair induced by the mobilization of stem cells besides other factors when the subchondral bone is opened. To imitate the process *in vitro*, a compression bioreactor dedicated to cultivate and remodel cartilage replacement material [21] has been modified allowing cell cultivation and load application in the same device.

This study aimed to introduce a novel bioreactor system *in vitro*, capable of inducing dynamic mechanical loading on a scaffold; and evaluate whether MSCs could shift from a compartment beneath to a scaffold after the mechanical stimulation, as cells might move when the subchondral bone is opened.

## Materials and methods

### Bioreactor specifications

**Fig 1:**
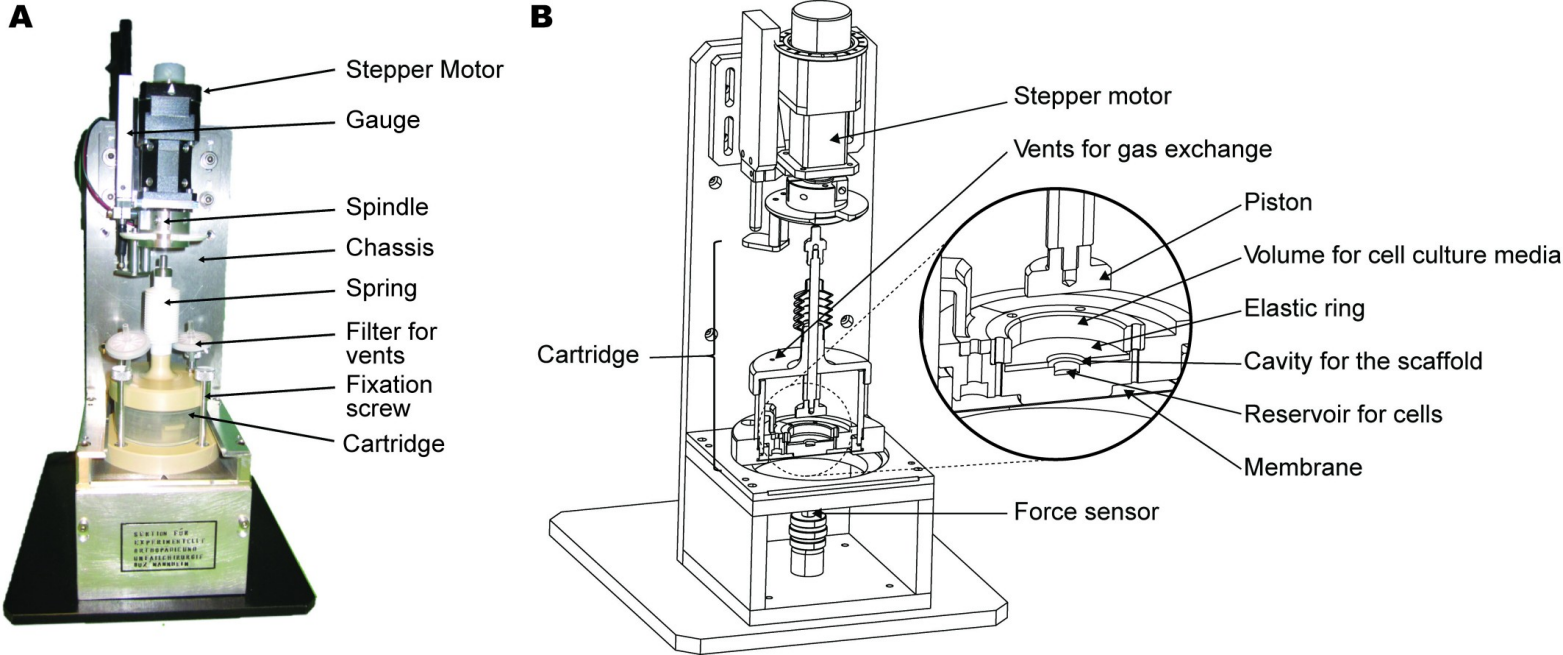
The Bioreactor. A) An authentic picture of the compression bioreactor is shown. A stepper motor drives the piston downwards and a spring (not visible) covered by a white bellows moves it upward. Parallel to the stepper motor, a digital gauge measures independently the vertical displacements of the piston. The cartridge is the housing for cell cultivation and mechanical application of the strain in the scaffold. It can be disassembled or assembled and hold to the bioreactor chassis by fixation screws. The cartridge cap comprises the embedded piston and the vents for gas exchange covered by 0.22 μm filters to protect from the external environment. B) The technical sketch depicts the inner structures of the bioreactor. The piston compresses simultaneously the elastic ring and the scaffold (not shown) located over the cell reservoir. A non-permeable membrane forms the bottom of the cartridge, isolating the cell cultivation system to protect it from contamination. A force sensor is located underneath the membrane, which is mandatory for reliable measurements of the induced forces. Dimensions of the bioreactor: 120 x 150 x 400 mm (width, depth, height), approximately.

**Fig 2:**
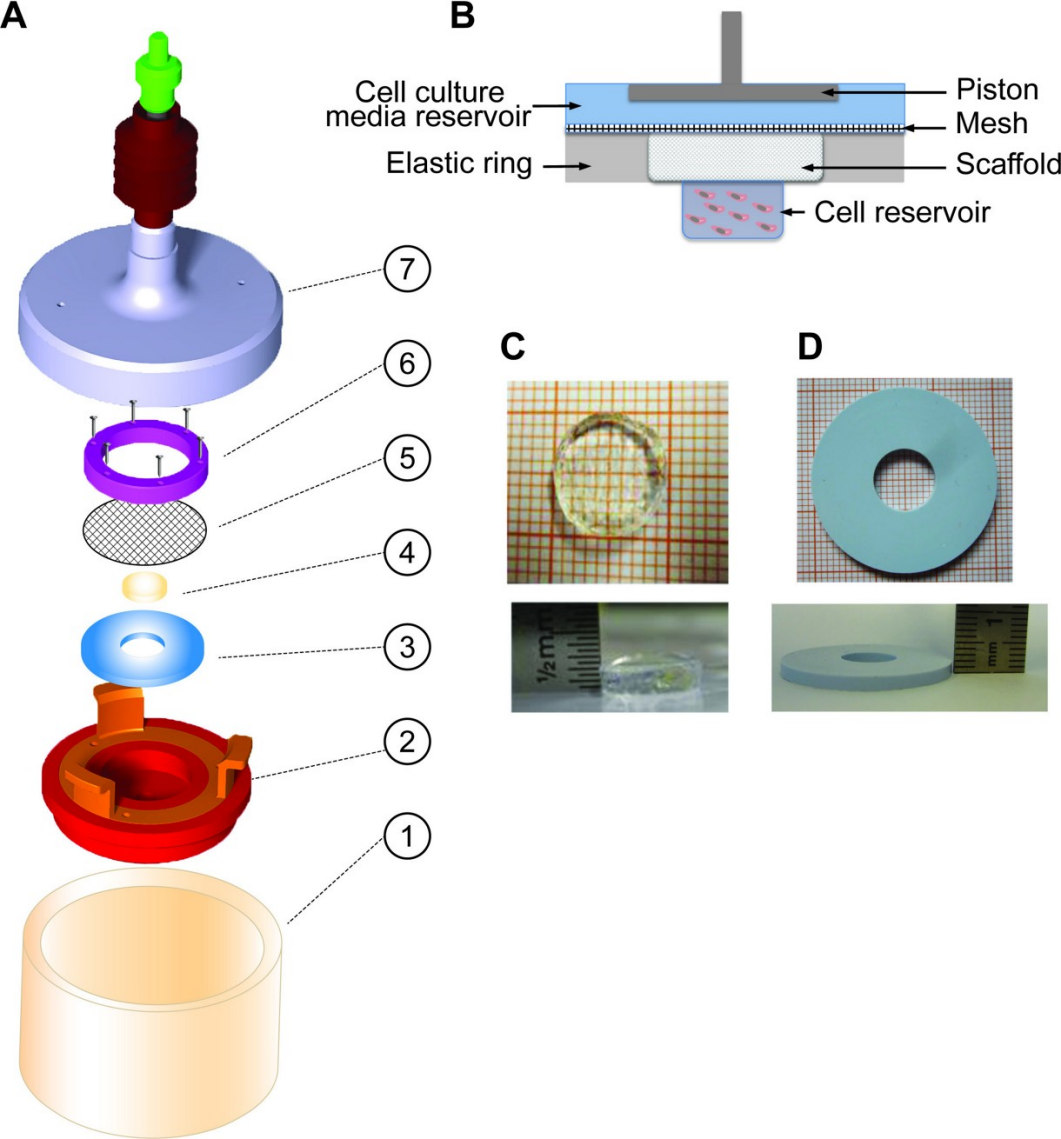
Assembling of the cartridge components. A) The sketch shows parts of a disassembled cartridge. The scaffold holder (2) contains the reservoir in the middle where the cells are placed. A scaffold (4) is placed over them, which is held in place by the elastic ring (3) and a mesh above it (5). The mesh is kept in place by a ring anchored to the construction using screws (6). The scaffold holder is placed as an independent movable unit within the cylindrical container (1) and it is covered up by a cap (7) containing the piston and spring. The cartridge is made of sterilizable materials. B) The schematic drawing shows the cross-section of the parts of an assembled scaffold holder without the upper anchored ring. The mesh aims to prevent the scaffold from moving up during the lift maneuvers. An alginate scaffold C) and the elastic ring D) are shown from above and a skew side view in detail.

A compression bioreactor was designed and built based on a model that was previously described [21] (Fig 1). To evaluate the shift of location of cells from one compartment to another in a 3D model, the bioreactor had to be completed with a lower chamber serving as cell reservoir, assembled below the target scaffold. The scaffold was surrounded by an elastic compressible ring of 2 mm height with an outer diameter of 30 mm and an inner diameter of 10 mm (Fig 2).

In the experiments, an upper positioned piston (20 mm Ø) was moved vertically downwards driven by a stepper motor (L4118S1404-T6x1-A25, Nanotec Electronic GmbH & Co, Feldkirchen, Germany) (Fig 1). The displacement of the piston induced compression acting simultaneously on the scaffold and the elastic ring. A force sensor (AUMMA-K001, Althen GmbH; Kelkheim, Germany), located underneath the cells reservoir measured the induced forces (Fig 1B).

The bottom of the cartridge was covered by a membrane (Permeaflon PTFE, Berghof Fluoroplastic Technology GmbH, Germany) to isolate the external environment and to communicate the displacements of the scaffold holder to the force sensor below via a rod (Fig 1B).

**Fig 3:**
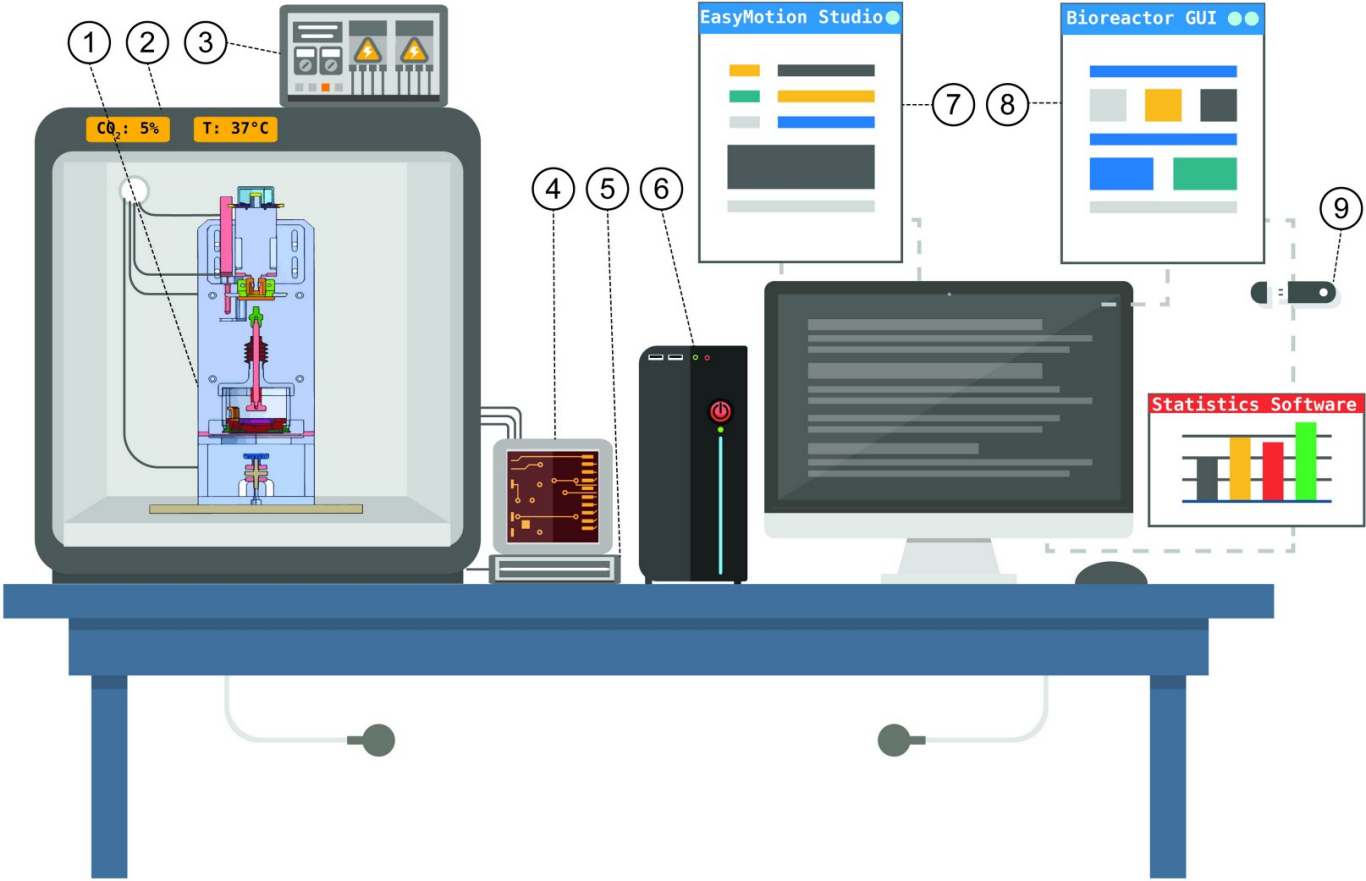
The system of the bioreactor is designed as standalone unit. The bioreactor (1) connected to a power supply (3) is placed inside a CO_2_ incubator to allow conditions for cell cultivation. The connection wires from the bioreactor are plugged over an electronic box (4) that contains a motion controller, an amplifier of the force sensor and the interpolator for the digital gauge. The motion controller receives the information from the stepper motor, and the signals of the force sensor generated by the loading are processed by the amplifier. The electronic box (4) is plugged to the connector block (5), which communicates with the PC card in the computer (6). The PC card synchronizes the beginning of the experiment with the motion controller software “EasyMotion” (7), where the settings are established for the experiments. Data as force, displacement of the piston and time are visualized and registered at a rate of 50 Hz by the custom-made software “Bioreactor”, programmed in LabView 2011. Finally, the data can be exported (9) and analyzed by statistical software such as Origin 9.0G, which was used in this study.

The compression bioreactor had to be dimensioned to be able to set it up in a CO_2_ incubator (Fig 3). Mechanical parameters of the experiments as the displacement were set by the custom-made software EasyMotion Studio (TECHNOSOFT SA, Neuchâtel, Switzerland), which handled the stepper motor through a motion controller (iPOS3604 MX Intelligent Drive, 144 W, CANopen / EtherCAT; Technosoft SA, Neuchâtel, Switzerland) (Fig 3). Right before starting an experiment, the motion controller synchronizes the information with EasyMotion software.

A digital gauge (DT32P, Sony, Tokyo, Japan) and its interpolator (MT11, Sony, Tokyo, Japan) were placed for measuring independently the vertical displacements given by the piston (Fig 1A), providing a resolution of 5 μm and an accuracy of ± 5 μm according to the information of the manufacturer.

The signals of the force sensor generated by the displacement were processed by an amplifier (SG-AP, Althen GmbH; Kelkheim, Germany). The amplified force signal was plugged to the connector block SCB-68A (National Instruments Corporation, Austin, Texas, USA), communicating the information to the PC-Card (PCI-6220, National Instruments Corporation, Austin, Texas, USA) for data acquisition (Fig 3).

Once the experiments were running, the synchronized data for the displacement of the piston, the forces, and time were visualized and registered by the custom-made data acquisition software “Bioreactor”, programmed in LabView 2011 (National Instruments Corporation, Austin, Texas, USA) at a rate of 50 Hz. The data were then exported and analyzed with the statistical software Origin 9.0G (OriginLab Corporation, Northampton, USA) (Fig 3).

### Cells isolation and cultivation

Porcine bone marrow derived-mesenchymal stem/stromal cells (pBM-MSCs) were isolated from femora of 3–5 days old pigs after being sacrificed for unrelated experiments (animal experimentation approval Nr. I-17/13, University of Heidelberg). For the hind limbs dissection, sterile instruments were used applying surgical technique. Briefly, skin, muscles, fat and connective tissue were removed. Then, the femora were immersed in 70% ethanol for 3 minutes. A transverse cut was made intercondylar with a scalpel and the bone marrow was scraped out with a surgical sharp spoon and harvested in 1X PBS with 1% penicillin/streptomycin. The suspension was centrifuged at 1000 rpm for 5 minutes and RBC lysis buffer was added for 5 minutes under constant stirring. The buffer lysis was removed by centrifugation at 1000 rpm for 5 minutes and 3 washes with 1X PBS were applied. The cells were then cultivated and expanded using supplemented-DMEM (10% FBS, 2 mM glutamine, 1% penicillin, and 1% streptomycin) at 37°C and 5% CO_2_, and 0.25% trypsin/EDTA was used for passaging. 500 cells per cm^2^ were plated in 75 cm^2^ cultivation flasks until the cells grew to ~80% confluence. pBM-MSCs were characterized at passage 1 by their fibroblast-like cell morphology and the surface markers CD44^+^ (eBioscience, Inc., San Diego, CA, US), CD90^+^ (Becton Dickinson, New Jersey, US), CD29^+^ (BioLegend, San Diego, CA, US), CD45^-^ (Becton Dickinson, New Jersey, US), SLA-1^+^ and SLA-DR^-^ (BioRad, Hercules, CA, US) by flow cytometry analysis (FACS Canto II, BD Biosciences). Passages 2 and 3 were used for the experiments.

### Scaffolds manufacturing

The target scaffolds of 10 mm in diameter and 2 mm in height (Fig 2C) were produced in a custom-made mold mixing 1.5% alginate (Keltone LVCR, ISP, US) diluted in 0.9% NaCl, with or without 15 μM LN521 (Biolamina; Stockholm, Sweden). Ionic polymerization of the alginate was induced by 0.1 M CaCl_2_ for 7 minutes.

### Silicone ring manufacturing

The elastic rings (Fig 2D) were made following the instructions of the manufacturer (Silicone Rubber high elasticity, Glorex AG, Füllinsdorf, Switzerland). Then, the silicone was cast in a custom-made plastic mold with the dimensions of 30 mm diameter, 2 mm height and a pin of 10 mm diameter in the middle. It was placed overnight for hardening at room temperature.

### Biomechanical stimulation

For the biomechanical examinations, 1.0x10^5^ pBM-MSCs were seeded in the cell reservoir in a mixture of supplemented-DMEM and 0.5% alginate, and covered by the scaffold, which was held in place by the elastic ring and a permeable mesh on top with a pore size of 160 μm (Nylon mesh 03-160/37, Plastok Associates Ltd, Birkenhead, UK). The reservoir had a volume of 42 mm^3^, approximately. Then, 2 mL of supplemented-DMEM were added over the scaffold. Unloaded scaffolds were used as controls prepared in parallel under the same conditions in another structurally identical cartridge.

Mechanical stimuli were applied as periodic dynamic compression exerted for 24 hours with an amplitude of 200 μm as 10% strain relating to the 2 mm height of the scaffold, and 0.3 Hz frequency. To allow nutrient and gas exchange, and waste disposal, the piston released the surface level of the scaffold and the elastic ring for 10 seconds each 180 cycles (i. e. after 10 minutes of loading) named as “lift” maneuver. To avoid hydrostatic disturbance between the gas atmosphere and the liquid during the unloaded interval, the released piston kept immersed in culture medium. The generated force by the applied periodical compression was continuously recorded for the whole duration of the test.

For executing the experiments, the starting position point of the piston was set manually by approximation until the piston touched the surface of the scaffold and the elastic ring, which was detected by checking the rise of the force value.

### Confocal microscopy

After 24 hours of dynamic load (or unload for controls), the scaffolds were stained with 7 μM calcein-AM and 5 μM ethidium-homodimer-1 for viability evaluation in 3D (Live/Dead kit, Invitrogen, Carlsbad, California, United States). Cells that mobilized into the scaffolds after stimulation were detected by confocal microscopy and counted by LAS X (Leica Application Suite X, Leica Microsystems) software. Viable and non-viable cells were identified by calcein-AM and ethidium homodimer-1 staining, respectively.

For the confocal microscopy, the scaffolds were placed in a custom-made holder. Then, DMEM-without phenol red was used as immersion media. 10 X magnification immersion objective, green and red channels (Alexa 488 for viable and Texas Red for non-viable cells, respectively) were used to visualize the cells. The whole scaffold was initially scanned manually to detect the cells located inside. 3D-images were taken with dimensions of 1.1 x 1.1 x 0.2 mm (length, width, depth) approximately. A resolution of 2048 x 2048 pixels (x and y axis, respectively) and 0.290 μm^3^ voxel size for 3D images were used in all cases at 600 Hz and bidirectional scanning (TCS SP8, Leica Microsystems, Wetzlar, Germany).

### Quantification of cells in 3D

The image analysis for the cells in the scaffold was done to detect and quantify the number of viable or non-viable cells present in each sample. The image analysis was done using Leica Application Suite X (LAS X) from Leica Microsystems. The ‘Analysis’ tab in the software GUI allows to create a custom image analysis pipeline using algorithms. Briefly, the applied pipeline consisted of 3D median filtering, manual thresholding, binary holes filling, 3D-image opening, binary image editing, and features calculation, as previously described [22]. Independent experiments for every condition were made in triplicates from the same donor.

### Viability tests of cells located in the reservoir

Viability of the cells located in the cell reservoir after the application of continuous or intermittent dynamic mechanical loading was measured by Trypan blue exclusion assay after 24 hours. Independent experiments were made in triplicates from the same donor.

### Statistical analyses

Statistical analysis was performed using Origin version 9.0G for Windows (OriginLab Corporation, Northampton, USA) to obtain descriptive statistics parameters as mean, standard deviation, median, maximum, and minimum.

**Fig 4:**
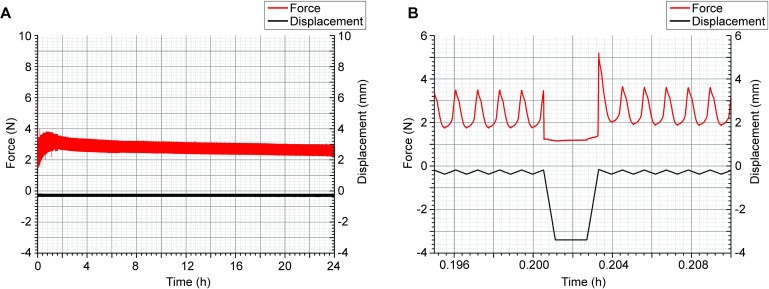
Recorded mechanical data after the test. A) Plot of the overview of a complete run. The force exerted over the system and detected by the force sensor is seen in red. The displacements of the piston detected by the gauge are seen in black. B) Lift maneuver. An unloaded phase of the intermittent dynamic mechanical loading is shown in detail. When the unloaded phase is reached, the piston moves upward (downwards in the depicted record as a black line). During the lift maneuver, the compression on the scaffold is lost and the basal force value corresponds to the offset. Note the elevated force peak when the piston compresses the scaffold again after the lift maneuver. In this case, an offset of about 1.2 N was seen during the lift maneuver.

The raw mechanical data obtained by Bioreactor GUI (National Instruments Corporation, Austin, Texas, USA) were exported, analyzed, and graphical charts of Fig 4 were prepared using Origin 9.0G. For the calculation of descriptive statistics of force-amplitude and displacement, the lifts maneuvers values were taken out.

**Fig 5:**
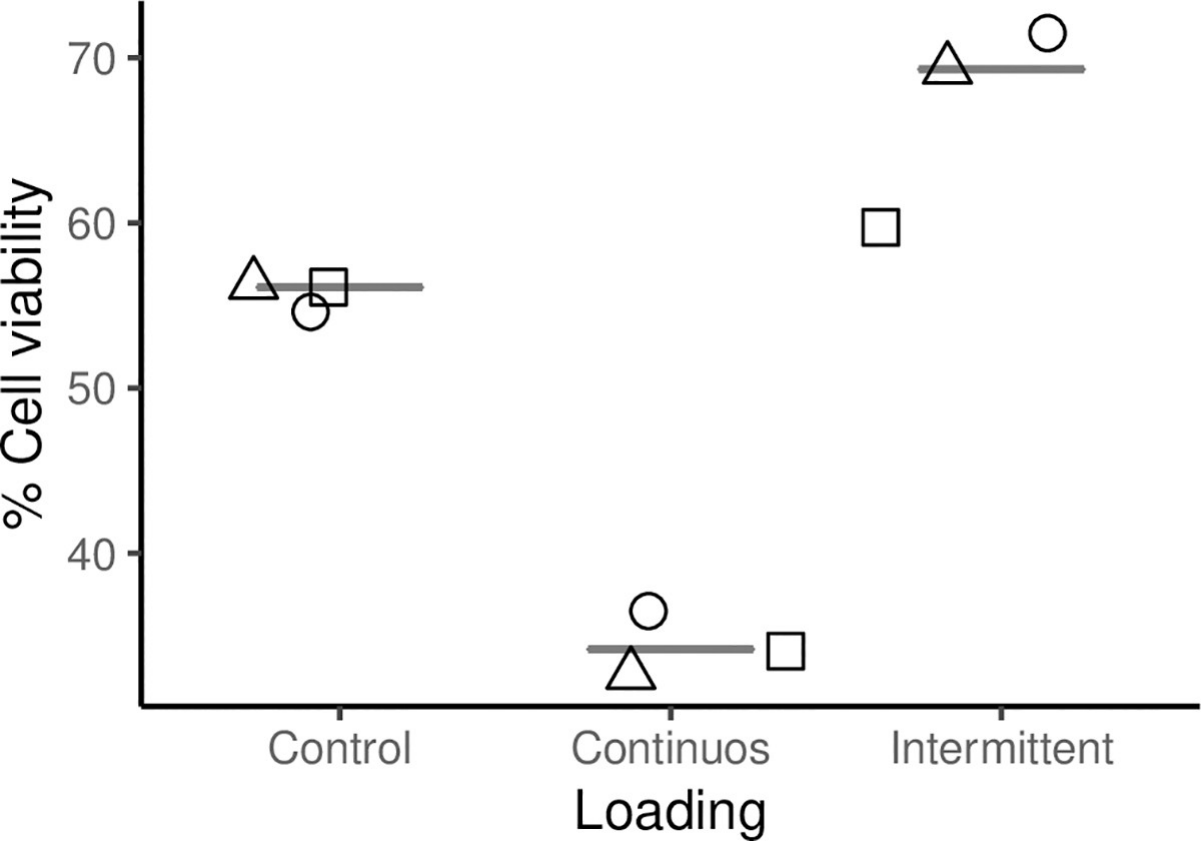
Viability of the cells residing in the reservoir. After 24 hours of mechanical stimulation, the viability of the cells which were not mobilized toward the scaffold was assessed by Trypan blue exclusion assay. The applied intermittent regime comprised 0.3 Hz frequency over 24 hours and interruptions with unloaded phases of 10 seconds after each 180 cycles. For the continuous regime, 0.3 Hz frequency for 24 hours without lift maneuvers loading was applied. The control scaffolds were prepared simultaneously in a separated and identically constructed cartridge, but no mechanical loading was applied. Continuous loading seemed to harm the cells, whereas interruption of the compression increased the cell viability comparable with unloaded cells. The results are shown as triplicates from the same donor; *Δ*, *Ο*, *□ = replicates*.

The cell viability values in the reservoir obtained by Trypan Blue assay, were processed in Origin 9.0G for descriptive statistics, and Fig 5 was prepared in R Core Team 2019 (Foundation for Statistical Computing, Vienna, Austria). Discrimination of replicates from the same donor was indicated by different symbols.

**Fig 6:**
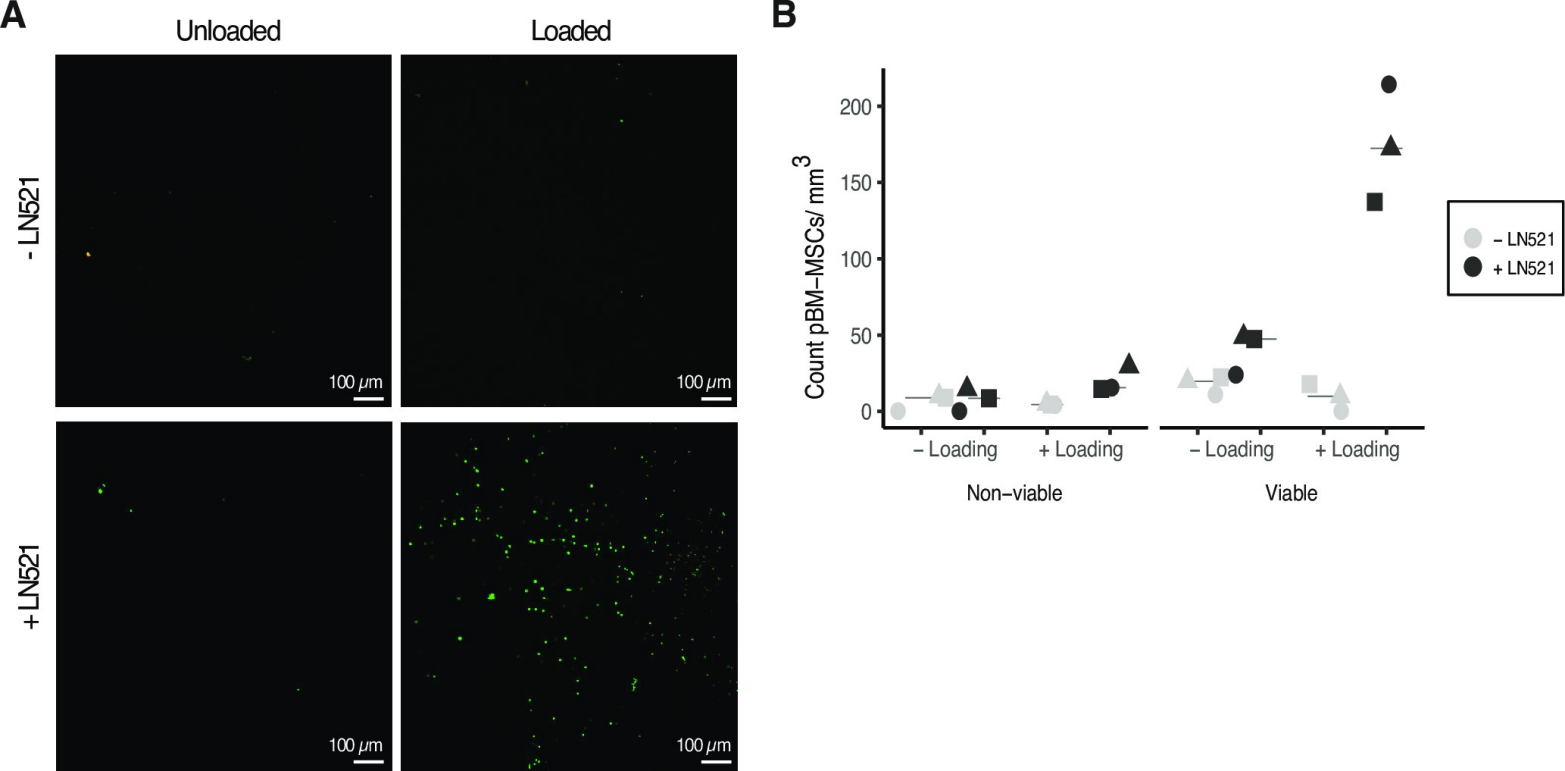
MSCs found in the scaffolds. A) Representative confocal microscopy images of alginate and alginate-LN521 scaffolds, with or without mechanical stimulation. Calcein-AM is seen in green and represents viable cells, ethidium homodimer-1 is seen in red and represents non-viable cells. There was only a low number of dead cells in the scaffolds. Bar scale = 100 μm. B) Quantification of viable and non-viable cells found in the scaffold after biomechanical stimulation or control. LN521 seems to have improved the alginate scaffold in terms of cell intake. The combination of LN521 in the scaffolds and the intermittent mechanical loading enhanced the number of cells found in the scaffolds. The results for every condition are shown as 3 independent replicates with cells from the same donor. Gray color indicates scaffolds without LN521 and black indicates the use of LN521; *Δ*, *Ο*, *□ = replicates*.

The count of viable and non-viable cells (presence or absence of loading or LN521) was obtained during the last step of the pipeline for quantifying cells in 3D in LAS X (Leica Application Suite X, Leica Microsystems) as an excel worksheet report. This information was used to calculate the counted cells per volume. Then, descriptive statistical analyses were carried out using Origin 9.0G, and Fig 6B was prepared in R Core Team 2019. Discrimination of replicates from the same donor was indicated by different symbols.

## Results

### Bioreactor

The redesigned bioreactor was able to induce mechanical loading on a biological arrangement composed by a scaffold and a reservoir of MSCs beneath it. It allowed us to evaluate the presence of cells in the scaffolds after mechanical loading stimulation. The cell cultivation system was provided as a cartridge (Fig 2) in a sealed design providing an aseptic environment for cell cultivation and the vents allowed gas exchange. Moreover, the cartridge was constructed as separate entity and could be easily handled, assembled and disassembled in a laminar flow bench.

The load transmission elements comprised the stepper motor driving the piston vertically downwards in a range from 0 mm (top) to 210 mm (bottom) and a spring moving it upward. When the spindle of the stepper motor was at the most upper position (0 mm), the cartridge could be easily mounted or taken out from the bioreactor chassis.

The scaffold holder (Fig 2A and 2B) contained a mesh on top overlapping the elastic ring, which kept the scaffold in place while the lift maneuvers were conducted. The elasticity of the ring apparently restored the shape of the scaffold after compression. In addition, the silicone material of the ring seemed to have sealed the reservoir and the upper chamber against liquid exchange under load.

The results showed that the bioreactor was able to stimulate the scaffolds over approximately 24 hours using a periodic regime with 0.3 Hz and resting time of 10 seconds each 180 cycles (Fig 4). The mean duration time for the 6 assessed independent examinations was 24.4 (±1.7) hours. No contamination of the cell culture was observed at the end of the experiments.

The values between the lift maneuvers starting after the first relief were taken into account for the calculation of descriptive statistics of the mechanical data as force-amplitude and displacement. The measured displacements were 185.8 (±17.8) μm in the mean for all 6 specimens. One specimen showed an irregularity as the curve of the recorded displacements revealed 2 steps over the running time, but the deviations acting on the scaffold were in the range from 155 μm to 175 μm (data not shown).

The calculated mean of the force-amplitude was 1.87 ± 1.37 N (min 0.31 N, max 4.42 N). The lifts of the piston for 10 seconds per cycle were carried out in the applied intermittent dynamic loading protocol and can also be seen in detail in Fig 4 with an estimated force offset of 1.2 N.

Furthermore, a single force-peak with a force interval of 3.03 ± 1.77 N (min 0.31 N, max 6.50 N) was observed in each cycle when the piston struck the surface of the scaffold once it had released (Fig 4B).

The scaffolds revealed no indications of heavy damage after the mechanical stress produced by the dynamic compression. A trace of stress was visible at the scaffolds after loading (S1 Fig) but not at the unloaded scaffolds used as control.

### Viability of the cells in the reservoir

We found that about 35% (median of 34.4%, ranging from 32.5 to 36.5%) of cells were viable after applying dynamic loading continuously without interruption (Fig 5). Under the intermittent dynamic load regime, we found a cell viability of about 70% (median of 69.3%, ranging from 60.0 to 71.1%). Moreover, the unloaded and intermittently loaded scaffolds acquired a reddish color, indicating that penetration and even distribution of cell culture medium in the scaffolds was allowed by the lift maneuver of the piston and it was independent of the exerted compression.

### Cells detected in the scaffolds

We found 13 ± 2 cells/mm^3^ (median of 13 cells/mm^3^, ranging from 11 to 15 cells/mm^3^, and 66.7% cell viability) in the scaffolds after 24 hours of mechanical stimulation, and 24 ± 11 cells/mm^3^ (median of 30 cells/mm^3^, ranging from 11 to 31 cells/mm^3^, and 73.7% cell viability) were present in the unloaded alginate scaffolds. However, when the scaffolds were supplemented with LN521, 48 ± 21 cells/mm^3^ were found in the unloaded scaffolds (median of 56 cells/mm^3^, ranging from 24 to 63 cells/mm^3^, and viability of 83.8%), and 194 ± 39 cells/mm^3^ (median of 202 cells/mm^3^ ranging from 152 to 230 cells/mm^3^, 89.7% cell viability) were present after loading (Fig 6).

In all cases, the cells in the scaffold were found in regions close to the adjacent cell reservoir.

## Discussion

Microfracture, ACI and mosaicplasty are the preferred treatments for AC defects, but those still fail to obtain a complete long lasting regeneration of AC [15]. One of the possible drawbacks to obtain a complete *in situ* regeneration of AC after microfracturing can be the lack of migration of enough number of progenitor cells resulting in fibrocartilage [23]. Therefore, we provide a tool where controlled variables can be evaluated *in vitro*, and a regime that suggests enrichment of cells in an acellular scaffold. The study provided herein suggests that intermittent biomechanical loading may induce the shift of location of cells from one compartment into a functionalized alginate scaffold against gravity, supporting our hypothesis that endogenous cells could be mobilized and recruited by biomechanical loading when the subchondral bone is opened. Our aim was to imitate *in vitro* a situation comparable when there is a defect in the cartilage layer of the tibia *plateau* of a knee joint by a modified bioreactor [21].

The relevant acting forces and amplitudes acting on the knee joint are hard to identify in terms of imitating the situation in bioreactors correctly, as they depend on the physical activity and conditions of the individuals. Thus, the compression generated on the AC by mechanical loading is more suitably referred as strain, which is defined as the change of the thickness regarding the original value of the height [24]. Daily normal activities have 0–10% strain, and 5–15% for post-activity; while 50–70% strain is reported to be injurious, and 70–90% to cause cell death [25]. In the tibiofemoral contact area, peak strains can be 7–23% during walking [25]. The strain evaluated in this study was comparable to diurnal cartilage strains at the medial tibia [24]. Parameters as frequency and strain were applied as previously reported [26], total time [27] and duration of unload phase [28] were between the ranges of previous studies.

The results indicate the necessity of functionalizing the scaffolds with LN521 to get more MSCs in, since the cells were not notably found in the scaffold made of only alginate (Fig 6). LN521 is a basement membrane protein present in stem cells niches, involved in cell adhesion, migration, and differentiation [29]. Therefore, structural proteins like LN521 may serve as adhesion factors for pBM-MSCs in the scaffolds. The functionalized scaffolds allow holding a higher number of MSCs after intermittent loading, supporting our hypothesis regarding mechanical stimulation.

Alginate is dense a material from which scaffolds can be made having a few μm of pore size [30]. A slight mark on the surface of the lower side of the loaded scaffolds was observed (S1 Fig), which corresponded to the circumference of the cell reservoir without apparent significant damage of the gel at microscopic level (S2 Fig). This side of the gel probably stretched toward the cell reservoir during loading. We can speculate that a modest change in shape of the loaded gels might cause an increase of the superficial area, promoting the adsorption/adhesion of cells on the scaffolds. However, it was the combination of LN521 and loading that resulted in more cells in the scaffolds.

Laminin cannot form hydrogels by its own due to the absence of electrostatic interactions or hydrogen bonding. However, it can be used within other hydrogels where functional groups may conjugate [31]. One of the limitations of our experimental setup is that we cannot be sure about the complete integration of LN521 into the alginate structure at molecular level, but we speculate that some of the LN521 molecules were trapped in the internal network of the scaffold. The use of oxidizing agents as IO4^-^ when preparing the scaffolds can be addressed for future approaches, allowing interaction between aldehyde groups of the oxidized alginate and amine residues of the laminin, as previously reported [32].

When the subchondral bone is perforated in a microfracture surgery, blood clots are formed and a suitable environment for tissue healing is created, promoting migration of MSCs and releasing growth factors and cytokines [33, 34]. The cultivation conditions in the cell reservoir given by a mix of culture medium and alginate solution increased the viscosity, which might have avoided cell sedimentation during the tests *in vitro* and helped with the shift of location of cells from one compartment to the other under loading stimulation.

For AC regeneration, studies applying either continuous [35, 36] or intermittent loading regime have been addressed by several authors in the past [37–39]. Cyclic is the more frequent loading pattern in the lower limbs joints during locomotion, where intermittent loading is part of the normal motion scheme since unloaded periods occur between load cycles [28]. From the mechanical point of view, the load pattern applied in this study showed a single higher peak of the force amplitude (Fig 4B) induced after the lift maneuver due to the piston moved downwards with a higher velocity after the released period. Thus, we found a complex loading pattern with interruptions and different forces acting on the scaffolds.

After the examinations with continuous dynamic loading, we found cell death at the cell reservoir, probably due to the lack of nutrients and gases exchange (Fig 5). The apparent increase of cell viability observed once the load was applied intermittently, suggests that nutrients and gas exchange may have occurred during the lifts of the piston. Therefore, the culture medium passed through the scaffold and reached the cells located at the cell reservoir, and the elastic ring prevented bypasses by a sealing effect under pressure. The applied intermittent dynamic mechanical stimulation seemed not to harm the cells when using functionalized scaffolds; in contrast, we observed a higher number of viable cells even after finding one value of the replicates close to the non-stimulated control (Fig 5). We would like to highlight that the cell reservoir had a limited size and thus, medium supply could affect viability within 24 hours. The viability of the cells in the control was 55% whereas corresponding viabilities in cell culture flasks were only slightly higher with 70% (data not shown). Hence, we speculate that the conditions in the cell reservoir were not optimal for 1x10^5^ cells as a volume of 80 μL of medium and alginate solution was limited, and the lack of nutrients and waste disposal may have harmed some cells for the observation time. However, the culture medium placed on top, and that reached the cell reservoir under intermittent loading, could have contributed to better cultivation conditions, resulting in 68% cell viability (Fig 5), which was close to the viability in cell culture flasks (data not shown).

To our knowledge, this is the first study suggesting mobilization of MSCs from a low compartment to another at the top against gravity, induced by biomechanical stimulation *in vitro*. Migration of chondrocytes under mechanical stimulation has been previously stated [40–42], and migration of MSCs under mechanical stimulation was addressed in the past by Ode and collaborators, demonstrating that loading hampered the mobilization of MSCs in bone healing context [43] using a bioreactor system previously described [44]. Both bioreactors were able to apply load on a scaffold. The main difference between them is that Ode at al., used a pneumatic force application system [44], whereas our force transmission was performed mechanically from a step motor over the stiff piston to the surfaces of the scaffold and silicone ring. The loading protocols also differ since Ode and collaborators applied 20% strain at 1 Hz for 72 hours, while our parameters were 10% strain at 0.3 Hz frequency as previously reported in [26], intermittently for 24 hours. The comparison of both approaches supports our assumption that mobilization of MSCs was not induced when applying mechanical loading alone (Fig 6). Our findings indicate that the cells were found in the hydrogel only when mechanical loading was applied intermittently and adjoined by the used of LN521-functionalized scaffolds.

Furthermore, higher levels of cell viability were found in the experiments performed using functionalized scaffolds, which indicates that LN521 may contribute to the wellness of cells during the test. We speculate that this explains why lower cell viability was found under loading in non-functionalized alginate scaffolds (Fig 6).

The experimental approach that we presented in our bioreactor has unique hardware and software architecture, composed of separated devices for cell cultivation, mechanical application, and software (Figs 1, 2 and 3). In addition, our focus was to analyze cartilage repair using functionalized-hydrogel scaffolds, where the cells were quantified by a custom made 3D-imaged based protocol [22].

This proof-of-concept study has some limitations. We presented 6 examinations, but it has to be taken into consideration that the cells were harvested from one animal. To be able to focus on the understanding of the mechanical behavior of the innovative device presented here, we controlled the amount of biological variation by not including MSCs from different donors. Thus, we refrained from further analytical statistical evaluation, and the biological variability will be addressed in a future study. Nevertheless, the effect on the number of cells found in the scaffolds was 4-5-fold higher after we applied intermittent mechanical stimulation, functionalized the alginate scaffold with the structural protein LN521, and dispersed the MSCs in the lower reservoir preventing cell sedimentation.

We detected the cells in the scaffold near to the surface of the scaffold that was close to the reservoir. At this time, we can only speculate how the cells shifted upward from the reservoir to the scaffold. At the current stage, we assume that this more likely happened because of external mechanical reasons rather than any biological active process from the cells as migration, as the shapes of the cells were quite spherical and specific tests for cell migration were not addressed in this study.

To explain the mechanisms of how the cells are transferred into the scaffolds is still challenging and opens new perspectives to be explored in detail in forthcoming research. To explore whether loading induces fluid movement from the lower compartment toward the scaffold, dark ink was placed at the cell reservoir in an early stage feasibility examination of the bioreactor. The ink diffused in the unloaded scaffolds showing a visible gradient, while fully stained the loaded scaffolds (S3 Fig). This result suggests that mechanical loading induces fluid to move through the scaffold. It is a matter of future studies to explain whether the cells were sucked-up or activated by other mechanisms to displace themselves. LN521 functionalization was important either to attract cells or to allow adherence of dispersed cells within the scaffold or both.

Future studies are necessary to find out whether the phenomenon suggested with these preliminary results will also be observed with cells from more than one donor, to assess human adult MSCs, to test different incubation times and to evaluate mechanical parameters that can increase the number of functional cells in the scaffolds.

With this bioreactor being a prototype to test our hypothesis of load inducing the mobilization of MSCs, the design is subject to improvements, e.g., self-replenishment of culture medium, scaffold composition, addition of supplementary factors in the culture medium for growth or differentiation.

Bone marrow is currently the most common MSCs source for cartilage regeneration research either alone or with biological scaffolds as so called matrix-augmented bone marrow stimulation [45]. Our experimental approach might be comparable to the *in vivo* situation when the subchondral lamella has been opened i.e., after microfracturing. As previously mentioned, current strategies for AC repair fail to produce hyaline cartilage. The presented bioreactor regime could provide new insights suggesting that endogenous progenitor cell mobilization to the defect site could be targeted by intermittent mechanical loading and functionalized-scaffolds. Moreover, mechanical loading is important in AC for distribution of nutrients, reinforce ECM content, fiber organization, and waste disposal [4, 5, 7]. The presented bioreactor system and preliminary examinations provide a first insight of loading probably involved in MSCs recruitment. An approach that achieves MSCs endogenous recruitment of MSCs, a proper combination of factors for chondrogenic differentiation, and phenotype maintenance would be beneficial for a fully functional regenerated AC.

Thus, the relevance of these findings for the orthopedic experimental research field is to establish a biomechanical system *in vitro* that gives us the chance to evaluate mechanical stimulation for cells moving from a compartment beneath, simulating MSCs moving from bone marrow *in vivo*. It may help afterwards to study molecular cues of mobilized *versus* non-mobilized cells and identify additional mechanical and chemical factors for inducing cells to be recruited by a given condition. Further steps will assess whether mechanical loading can induce MCSs for bone or cartilage differentiation. Nonetheless, the first results of the presented study suggest that mechanical stimulation may have an important impact on the mobilization of stem/stromal cells. Thus, a combination of the current strategies applied for AC-regeneration as microfracture, biomechanical protocols and functionalized scaffolds might enhance the outcomes of the current treatments applied in osteoarthritis or AC-trauma.

## Conclusions

We present a bioreactor system for mechanical load application on an *in vitro* biological arrangement as scaffolds. The preliminary results suggest that the stimulation exerted by the applied intermittent dynamic loading protocol, combined with LN521 functionalization of the target scaffold can promote the mobilization of pBM-MSCs from a lower compartment toward the scaffold in the compartment above, against gravity.

## Supporting information

S1 Fig Stress on the scaffold after the mechanical stimulation. Once the scaffold was loaded for 24 hours, a trace of mechanical stress was visible as an inner circumference, which corresponds with the cell reservoir edges.
(TIF)

S2 Fig Z-stack of LN521-funtionalized scaffolds. 3D images of the region of interest, corresponding to the scaffold side adjacent to the cell reservoir are shown. The cells present in the loaded scaffold suggest that the scaffold surface did not suffer substantial damage after mechanical stimulation.
(TIF)

S3 Fig Mechanical loading enhances the fluid uptake. Dark ink was placed in the cell reservoir compartment to explore whether loading modified the influx of the fluid. The control shows that the ink diffused into the scaffold showing an evident gradient, whereas loaded scaffold was totally stained. This suggests that mechanical loading induces fluid to move toward the scaffold.
(TIF)

S1 Table Raw data excel file. Sheet1) Mechanical stimulation data of every replicate with loading or no loading for controls, and a summary of the statistics are shown in the worksheet named “Biomechanical_data”. Sheet2) The viability of cells after the mechanical stimulation located at the cell reservoir and in the scaffold is shown in the worksheet named “Cell_viability”. Sheets3-14) The data obtained after counting the cells in 3D using LAS X software are shown in the worksheets 3 to 14, named by experimental condition. The counts of viable and non-viable cells can be seen in channels 1 and 2, respectively.
(XLSX)
