# Supplementary figures and images for: Bioreactor for mobilization of mesenchymal stem/stromal cells into scaffolds under mechanical stimulation: Preliminary results
Source: PLoS One. 2020 Jan 10;15(1):e0227553. doi: 10.1371/journal.pone.0227553 (PMC6953860; doi:10.1371/journal.pone.0227553)

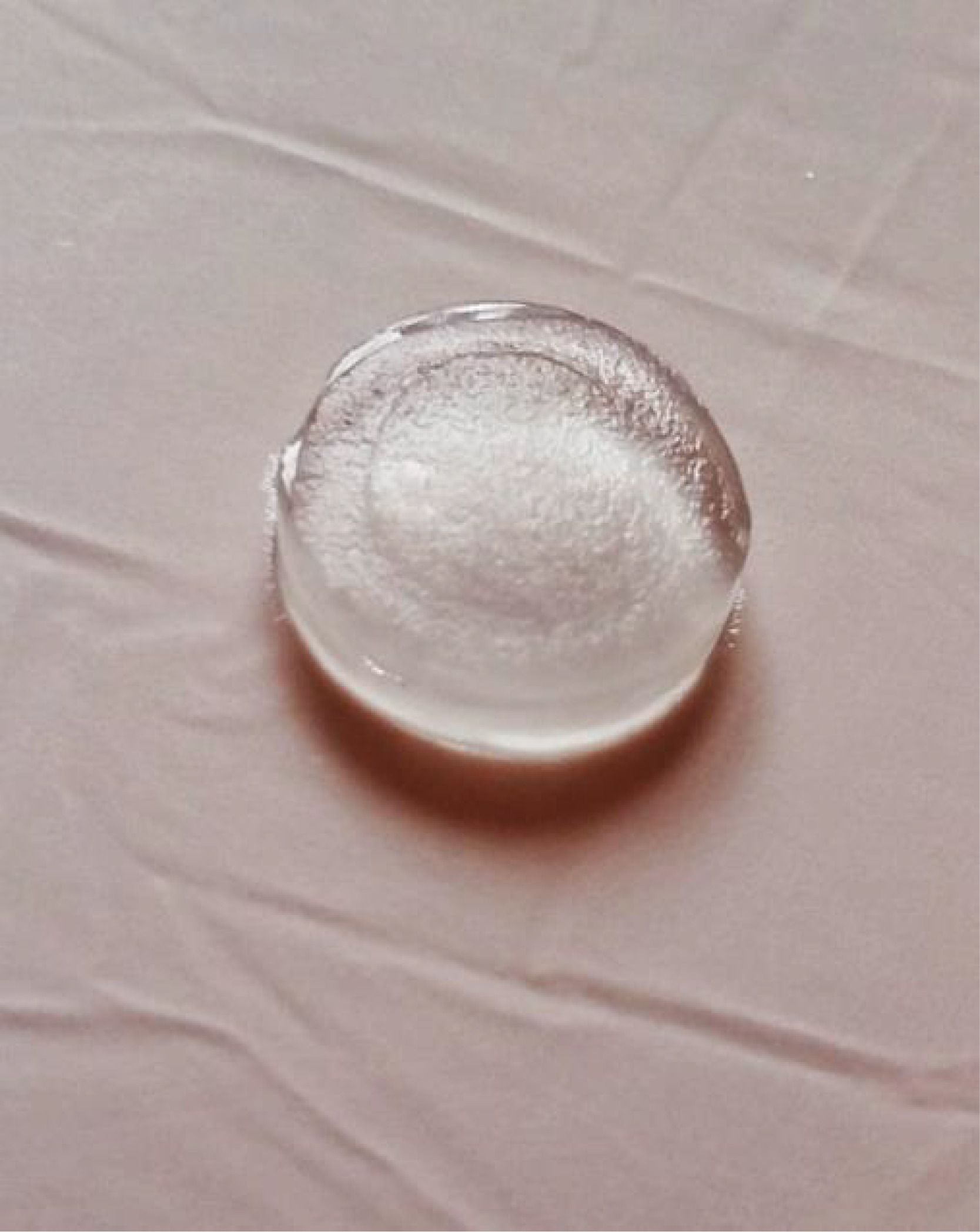

Supplement: S1 Fig — Once the scaffold was loaded for 24 hours, a trace of mechanical stress was visible as an inner circumference, which corresponds with the cell reservoir edges. (TIF) [file pone.0227553.s001.tif]

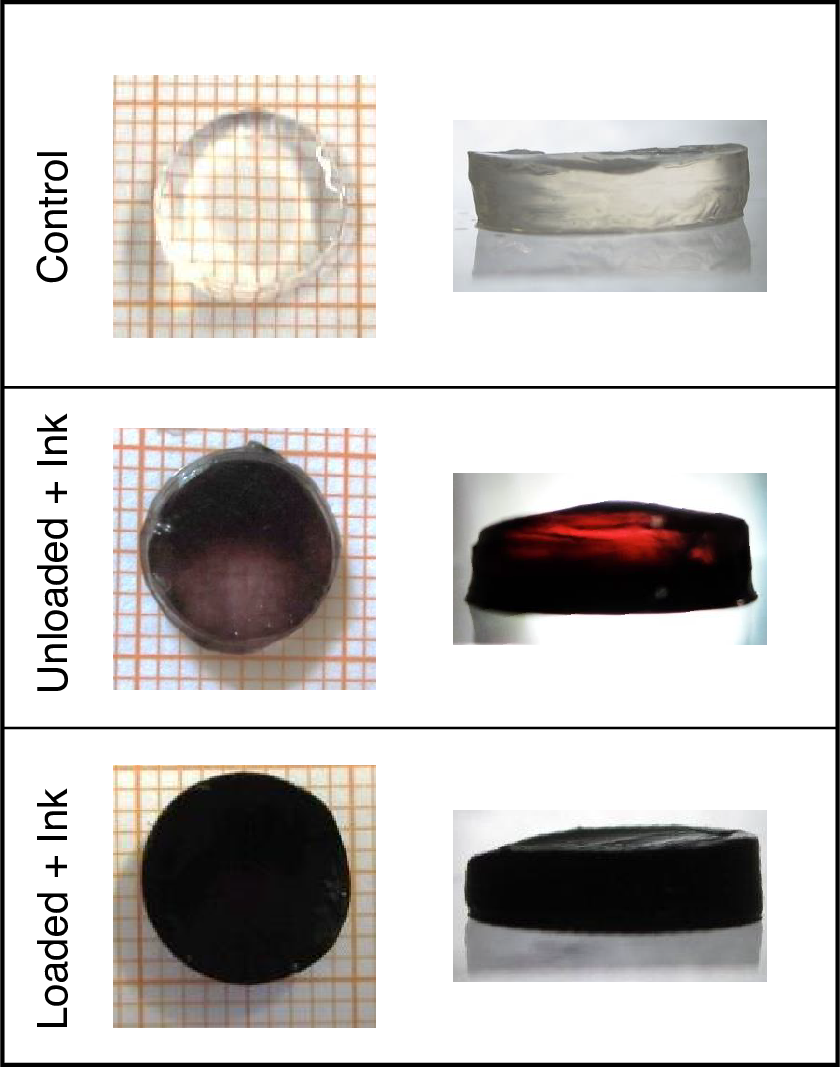

Supplement: S2 Fig — 3D images of the region of interest, corresponding to the scaffold side adjacent to the cell reservoir are shown. The cells present in the loaded scaffold suggest that the scaffold surface did not suffer substantial damage after mechanical stimulation. (TIF) [file pone.0227553.s002.tif]

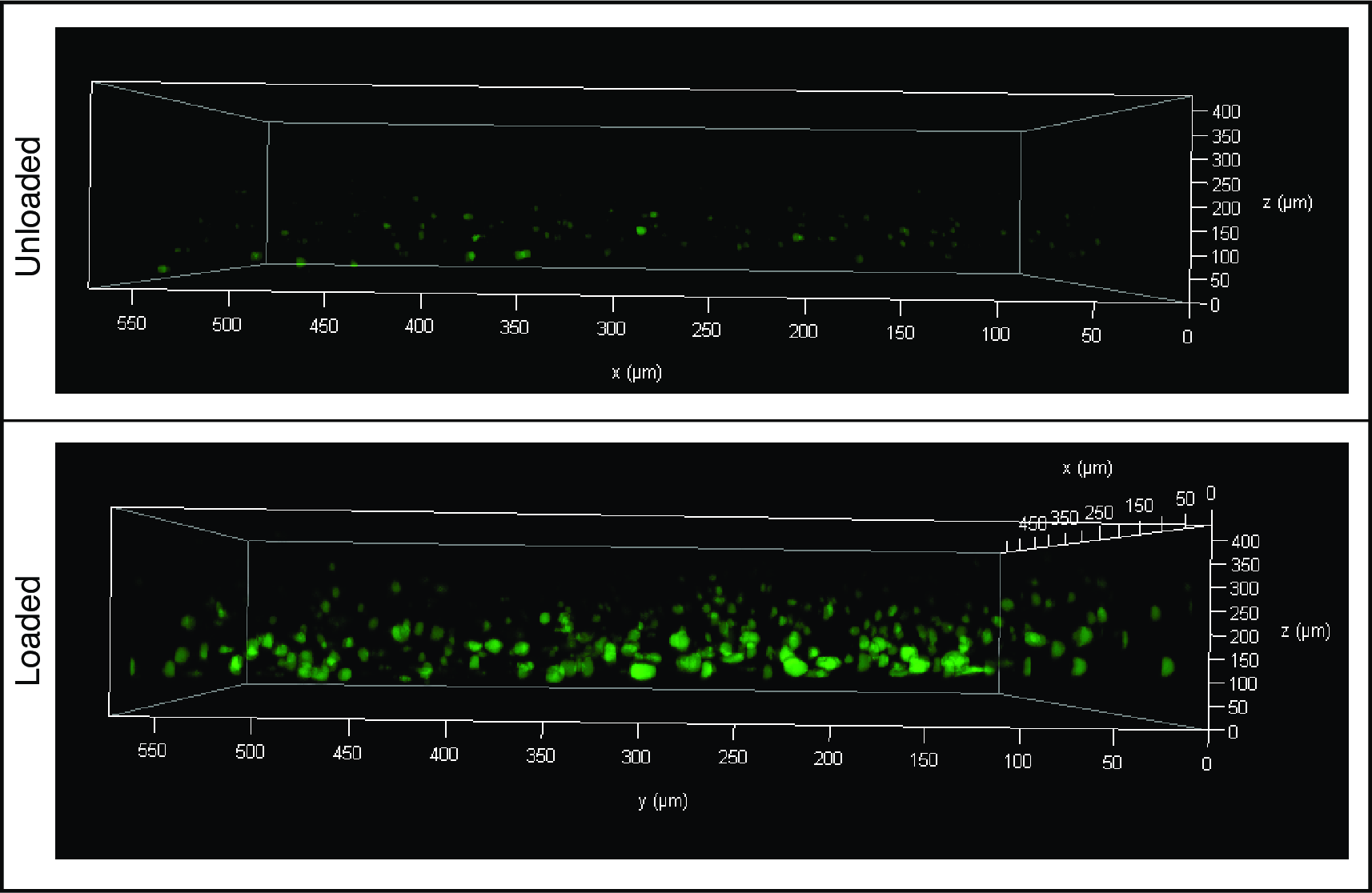

Supplement: S3 Fig — Dark ink was placed in the cell reservoir compartment to explore whether loading modified the influx of the fluid. The control shows that the ink diffused into the scaffold showing an evident gradient, whereas loaded scaffold was totally stained. This suggests that mechanical loading induces fluid to move toward the scaffold. (TIF) [file pone.0227553.s003.tif]
